# Low-tube-voltage combined with adaptive statistical iterative reconstruction-V technique in CT venography of lower limb deep vein thrombosis

**DOI:** 10.1038/s41598-018-29519-y

**Published:** 2018-07-24

**Authors:** Dan Chen, Jiahui Zhou, Peixi Wang, Quanxu Ge, Min Xu, Wei Qiu, Xinnan Li, Xiaodong Wang

**Affiliations:** 1 Department of Radiology, Weihai Municipal Hospital, Weihai, 264200 Shandong China; 2 Department of Anesthesia, Weihai Municipal Hospital, Weihai, 264200 Shandong China

## Abstract

This study contains 2 arms: (1) the ASIR-V technique combined with low-tube-voltage in lower limb deep vein thrombosis (DVT) diagnosis was investigated; and (2) CT venography and ultrasound results in DVT diagnosis were compared. For arm 1, 90 patients suspected of DVT were randomly divided into 3 groups (30/group): groups A and B were scanned under 100-kV with pre-set ASIR-V weights of 30% and 50% respectively; group C were scanned under 70-kV with a 50% weight. For arm 2, 75 patients were divided into 3 groups (25/group), each group was CT scanned as in arm 1 and then all subjects were examined by ultrasound. Groups A, B and C had 16, 14 and 17 patients diagnosed with DVTs, respectively. There was no significant difference in subjective ratings of image quality among all groups. The 70-kV protocol remarkably increased venous attenuation value while all groups had similar DVT attenuation value. Higher noise was observed in group C, the CNR however, was actually augmented due to elevated venous attenuations. More importantly, group C had significantly lower CTDI_vol_ and DLP values. In conclusion, the 70-kV protocol is superior to the 100 kV protocols, which was supported by findings from the second arm study.

## Introduction

Deep vein thrombosis (DVT), the formation of blood thrombi in the deep veins, remains a common and serious clinical condition worldwide. It often manifests as DVT in the lower extremity, but may also occur in veins of other body parts, e.g., in cerebral sinus, arms, retina, etc^1,2^. DVT in the lower extremity can progress to involve the proximal veins, leading to pulmonary embolism^3–5^, a life-threatening condition with a mortality rate of 2–3%^6^. DVT can effectively be treated with anticoagulants, thrombolytic agents or embolectomy^1,7,8^, underscoring the importance of early and accurate diagnosis of DVT.

As DVT is often asymptomatic and its clinical features are unspecific, to date, diagnosis of DVT relies on imaging technologies, such as ultrasound and computed tomography (CT) venography^9^. It has been shown that CT venography is superior to ultrasound in the detection of DVT in calf veins, and in identification of pelvic or abdominal thrombi^9–11^. Currently, radiation exposure still remains a serious concern in CT scanning^12,13^. A variety of techniques beyond the traditional reconstruction method of filtered back projection (FBP) have evolved, such as hybrid iterative reconstruction, model-based iterative reconstruction (MBIR) and adaptive statistical iterative reconstruction (ASIR). The use of advanced reconstruction techniques allows for low-dose protocols, since they yield higher quality imaging data when compared to FBP^14–17^. Recently, a new generation of ASIR technique, namely ASIR-V, has emerged and been compared with other analytical reconstruction methods, e.g., FBP, MBIR and ASIR^18–21^. The effects of low-tube-voltage in conjunction with ASIR-V in CT venography for DVT has not been investigated. This study was therefore conducted to compare the impacts of 70 kV and 100 kV combined with ASIR-V technique in CT venography of lower limb DVT.

## Materials and Methods

### Patients

The protocol of this prospective study was reviewed and approved by the Research Ethics Committee of Weihai Municipal Hospital. This study was conducted in accordance with the guidelines of biomedical research involving humans established by the Ministry of Health of the People’s Republic of China. Informed written consent to participating in this study was obtained from all subjects. This study contained 2 arms, the first was to examine the effect of different CT protocols. For this purpose, 90 patients suspected of DVT were recruited. The second was to compare the CT venography and ultrasound results in detection of DVTs, and additional 75 patients were enrolled. All patients enrolled did not have severe liver, kidney or cardiovascular disease. Those who were allergic to iodine contrast media or with a body mass index ≥30 were excluded.

### Arm 1: CT venography

ASIR-V still uses FBP data sets as building blocks for image reconstruction and is blended with FBP in 10% increments. A higher percentage of ASIR (weight 60–70%) results in degradation of image quality^18,22^. Therefore, a weight of 50% was chosen as the maximum ASIR-V blending percentage in this study. For the first arm of study, 90 patients were randomly divided into 3 groups (30 subjects/group): patients in groups A and B were scanned by GE Revolution CT under a tube voltage of 100 kV with pre-set ASIR-V weights of 30% and 50% respectively, and a noise index of 10 (the tube current was automated between 0–400 mAs). Patients in group C (with a pre-set ASIR-V weight of 50%) were scanned with the same parameters as those in groups A and B, but with a tube voltage of 70 kV and an automated tube current between 0–500 mAs. The contrast medium, lopromide, was injected (1.5 ml/kg) at the speed of 3.5 ml/s in the right antecubital vein followed by the injection of 60 ml saline. Three minutes post the administration of saline, CTV was performed from pelvis to feet following routine clinical practice (a gantry rotation time of 0.35 seconds with a pitch of 0.992). Images were reconstructed using a section thickness of 5.0 mm without overlap.

### Arm 1: Image post-processing and reconstruction

Image post-processing and reconstruction were accomplished on the GE ADW4.6 workstation using maximum intensity projection, multi-planar reformation and volume rendering techniques.

### Arm 1: Quantitative image analysis

Objective image analysis was done by measuring the attenuation (in HU) in the inferior vena cava, iliac vein, femoral vein, and popliteal vein at the L4 vertebral body, the inferior margin of the sacroiliac joint, the greater trochanter of the femur, and the medial epicondyle of the femur, respectively. A small round region of interest (ROI) was set at the center of the vein lumen for such measurement. To quantify the attenuation differences between the vein and the surrounding tissue, the attenuation of psoas, iliopsoas, iliacus and semimembranosus muscles was also measured. The image noise was determined as the standard deviation of the attenuation in the muscles. Contrast-to-noise ratio (CNR) was calculated using the formula: (the attenuation value of the vein - the attenuation value of the adjacent muscle)/noise. Similarly, the attenuation value of DVT was measured by placing a small oval ROI in the DVT. If multiple DVTs were detected, the largest was selected for measurement.

### Arm 1: Qualitative image analysis

Qualitative image analysis was performed independently in a blinded manner by 2 radiologists who had over 5 years of experience in CT venography interpretation. Images were evaluated for graininess, streak artifact and vessel enhancement. Overall image quality was rated using a 4-point scale as described elsewhere^17^, with 4 being the highest score, indicating an excellent diagnostic value, and 1 the lowest score, indicating that the image is uninterpretable.

### Arm 1: Analysis of radiation dose

The volume CT dose index (CTDI_vol_) and dose length product (DLP) automatically recorded by the CT scanner after each scanning were used for the analysis of radiation dose.

### Arm 2: Comparison of diagnostic results between CT venography and ultrasound scanning

In this arm of study, 75 patients were randomly divided into 3 groups (25/group): patients in group 1 were scanned using the 100-kV with 30% weight protocol; group 2 using 100-kV with 50% weight and group 3 using 70-kV with 50% weight as described above. Additionally all patients were also examined with ultrasound. Compression and pulsed wave Doppler ultrasound were performed by 2 technologists with over 10-year experience using the iU22 ultrasound system from Philips (Amsterdam, Netherland) with a 5–12 MHz transducer. Compression was performed at 2-cm intervals from the inguinal ligament to the ankle.

### Statistical analysis

Data normality was determined by the Shapiro-Wilk test. All continuous data were expressed as mean ± standard deviation, and analyzed by one-way ANOVA with Fisher’s least significant difference post hoc test. Categorical data were tested with the Chi-square test. Inter-rater agreement between the 2 radiologists was determined by Cohen’s kappa test. Interpretation of the Cohen’s kappa coefficient K value was as follows: K > 0.75, good agreement; 0.4 < K < 0.7, moderate agreement; and K < 0.4, slight agreement. All statistical analyses were performed using the SPSS 20.0 software and a p-value of <0.05 was considered as statistically significant.

## Results

### Arm 1: Patients

**Figure 1:**
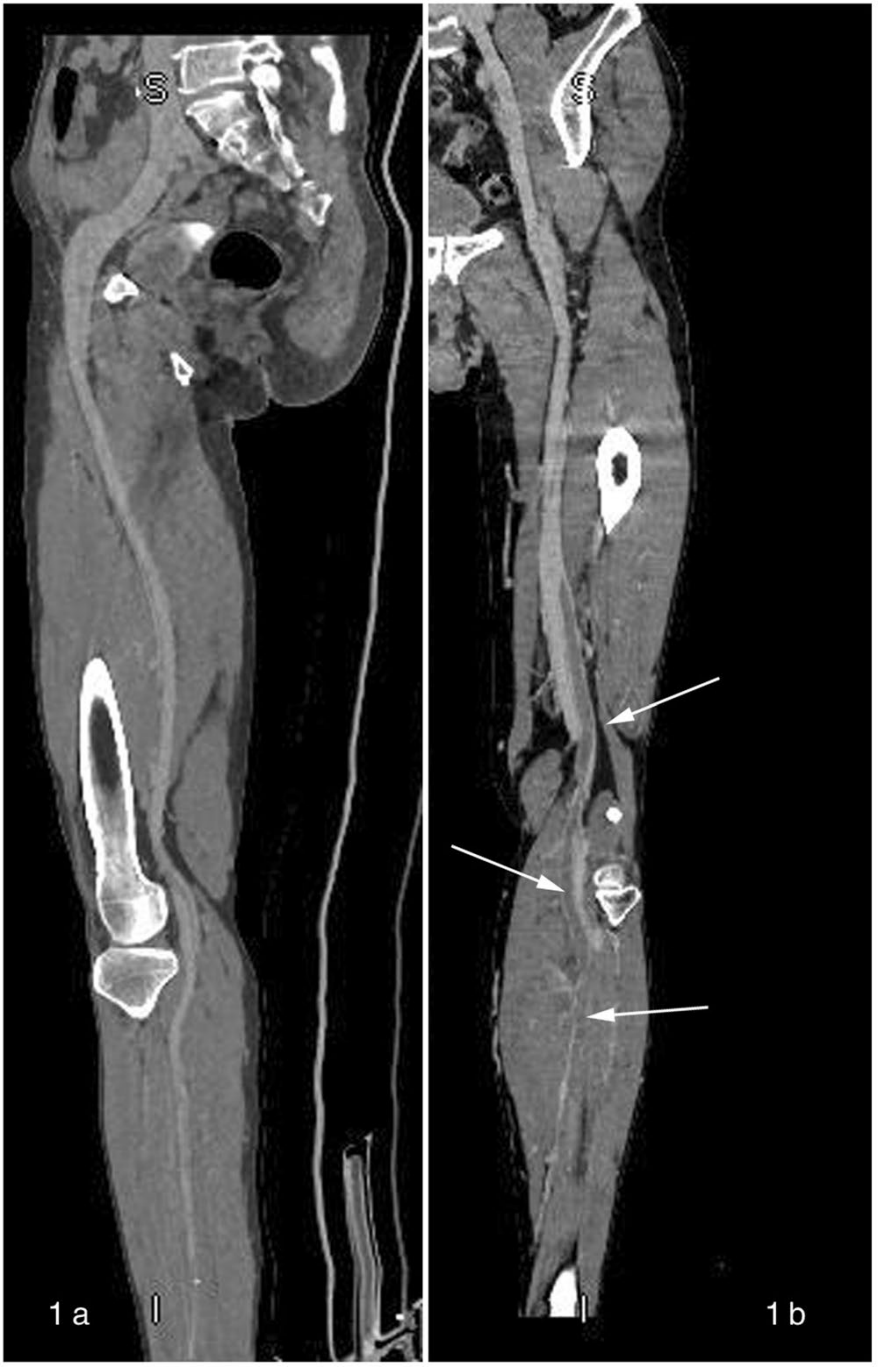
CT venography of normal veins and DVTs. Veins in panel 1a are normal, while multiple DVTs are seen in panel 1b (intraluminal filling defects indicating DVTs are shown by the arrows).

Demographics and basic characteristics of all patients are shown in Table 1. There were no significant differences in age, gender ratio, weight, height or BMI among all groups of patients. Groups A, B and C had 16, 14 and 17 patients diagnosed with DVT by CT venography, respectively (The diagnosis of DVT was made after clots were identified in CT images, see Fig. 1).

**Table 1:** Patients’ demographics and basic characteristics in arm 1 study.

	Group A	Group B	Group C	P value
Age	54.37 ± 13.12	57.03 ± 12.64	55.40 ± 12.26	0.715
Sex (M/F)	19/11	22/8	21/9	0.696
Height (cm)	170.9 ± 7.2	171.1 ± 7.3	172.3 ± 6.6	0.883
Weight (kg)	73.9 ± 9.4	72.6 ± 8.1	73.2 ± 10.7	0.862
BMI (kg/m^2^)	25.3 ± 2.5	24.7 ± 1.6	24.7 ± 2.2	0.495

M: male; F: female; BMI: body mass index. Continuous data were expressed as mean ± standard deviation and tested by one-way ANOVA. The categorical data (sex) were analyzed by the Chi-square test.

### Arm 1: Quantitative image analysis

**Figure 2:**
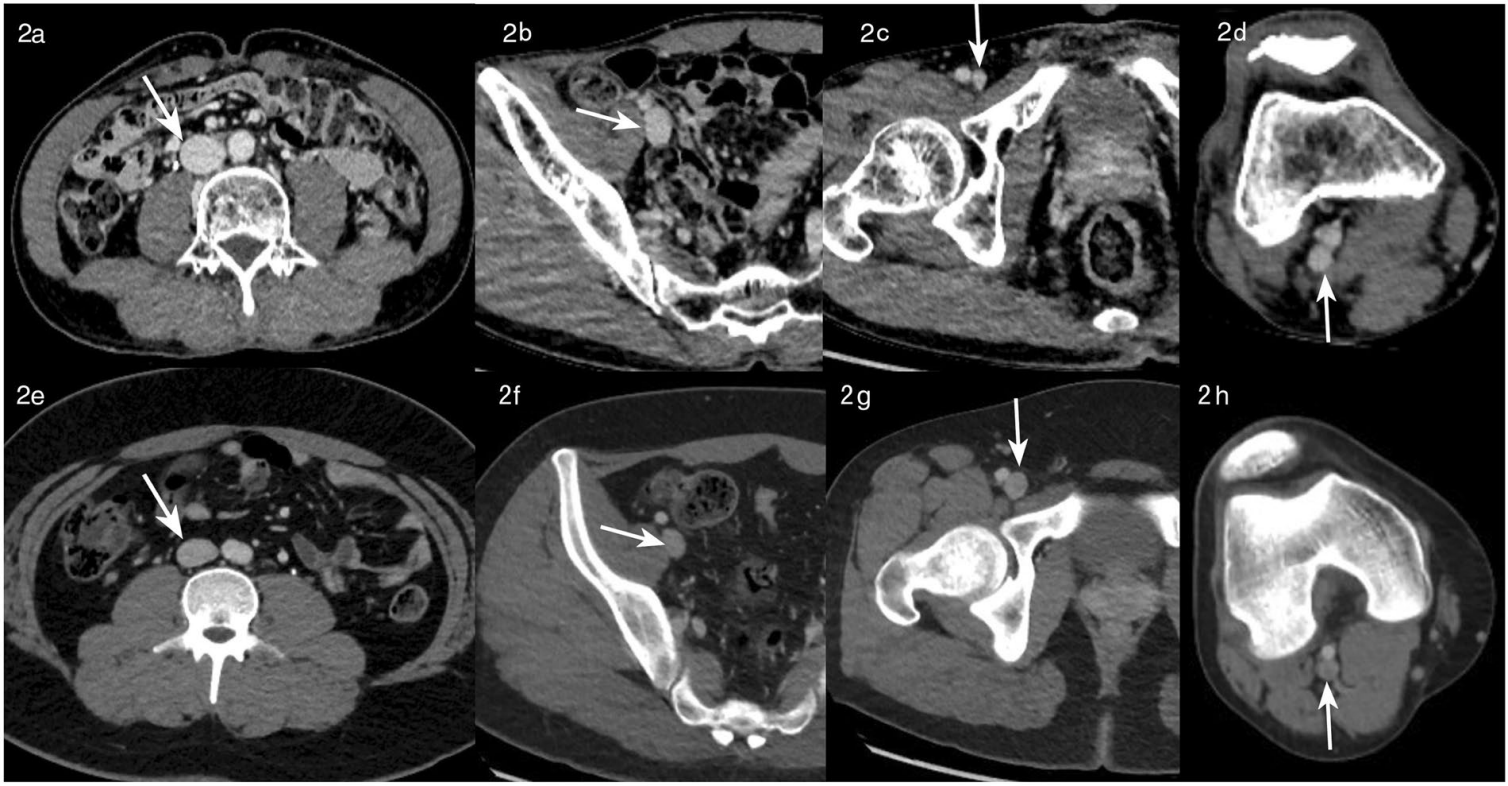
Venous enhancement under 70 kV and 100 kV. Representative images under 70 kV: inferior vena cava (**2a**) right iliac vein (**2b**) right femoral vein (**2c**) and right popliteal vein (**2d**). Representative images under 100 kV: inferior vena cava (**2e**) right iliac vein (**2f**) right femoral vein (**2g**) and right popliteal vein (**2h**). Venous enhancement (arrows) was stronger at all levels under low-tube-voltage.

The mean attenuation values of all measured veins in groups A, B and C were 113.23 ± 11.98 HU, 119.16 ± 12.89 HU and 157.90 ± 21.78, respectively, of which, no significant difference was observed between groups A and B. However, group C had a markedly higher mean attenuation value than groups A and B (both p < 0.001, Table 2). Representative images from groups B and C demonstrating the venous enhancement are shown in Fig. 2 (venous enhancement was stronger at all slice levels under 70 kV). There was no significant difference in the average noise between groups A and B. In contrast, group C had higher average noise compared with groups A and B (Table 2). Nevertheless, the average CNR in group C was significantly higher than those in groups A and B (Table 2). Similar results were observed in all measured segmental veins, i.e., in inferior vena cava, iliac vein, femoral vein and popliteal vein (Table 2). The mean attenuation values of DVT in groups A, B and C were 65.71 ± 3.63 HU, 65.53 ± 3.65 HU and 66.43 ± 3.45 HU, respectively. There were no significant differences in mean attenuation values of DVT among the 3 groups.

**Table 2:** Computed tomography venography data.

	Group A	Group B	Group C
Inferior vena cava			
Attenuation value	124.99 ± 14.45	132.57 ± 13.51	172.20 ± 23.19*
Noise	16.75 ± 4.05	17.17 ± 3.39	19.98 ± 3.36^#^
CNR	3.38 ± 1.30	3.94 ± 0.98	5.17 ± 1.36*
Iliac vein			
Attenuation value	119.69 ± 15.17	123.83 ± 17.87	164.57 ± 27.87*
Noise	17.49 ± 3.79	17.38 ± 3.63	20.17 ± 5.83^∆^
CNR	3.36 ± 1.02	3.74 ± 1.05	4.91 ± 1.41*
Femoral vein			
Attenuation value	113.47 ± 12.45	119.28 ± 15.61	161.51 ± 27.31*
Noise	15.49 ± 4.38	15.83 ± 4.09	18.88 ± 6.42^∆^
CNR	3.60 ± 1.23	3.99 ± 1.51	5.29 ± 1.78*
Popliteal vein			
Attenuation value	94.78 ± 11.41	100.96 ± 13.83	133.32 ± 23.99*
Noise	9.32 ± 12.57	10.06 ± 2.36	11.53 ± 2.28^∆^
CNR	5.39 ± 1.76	5.45. ± 2.15	7.06 ± 2.69^**$**^
Mean of all veins			
Attenuation value	113.23 ± 11.98	119.16 ± 12.89	157.90 ± 21.78*
Noise	14.76 ± 2.96	15.11 ± 2.04	17.64 ± 3.40*
CNR	4.14 ± 1.03	4.32 ± 1.03	5.71 ± 1.46*

CNR: contrast-to-noise ratio. *P < 0.001 compared to both groups A and B; ^#^P < 0.005 compared to both groups A and B; ∆ P < 0.05 compared to both groups A and B; and ^$^P < 0.01 compared to both groups A and B. All data were expressed as mean ± standard deviation, and analyzed by one-way ANOVA with Fisher’s least significant difference post hoc test.

### Arm 1: Subjective image analysis

The Cohen’s kappa test showed that the rating of images between the 2 radiologists was in good agreement with a K = 0.78. In a 4-point scale analysis, the overall image scores in groups A, B and C were 3.20 ± 0.52, 3.19 ± 0.50 and 3.34 ± 0.58, respectively, which are not significantly different (Table 3). Scores of pelvic and thigh regions among the 3 groups were similar (Table 3). However, the score of the leg was significantly higher in group C compared with those in groups A and B (both p < 0.05, Table 3).

**Table 3:** Scores from subjective analysis of images.

	Group A	Group B	Group C
Pelvic	3.42 ± 0.56	3.37 ± 0.60	3.45 ± 0.62
Thigh	3.45 ± 0.55	3.40 ± 0.62	3.43 ± 0.63
Leg	2.75 ± 0.76	2.77 ± 0.70	3.15 ± 0.62^∆^
Overall	3.20 ± 0.52	3.19 ± 0.50	3.34 ± 0.58

^∆^P < 0.05 compared to both groups A and B. All data were presented as mean ± standard deviation, and analyzed by one-way ANOVA with Fisher’s least significant difference post hoc test.

### Arm 1: Radiation dose

Radiation dose data are shown in Table 4. The CTDI_vol_ (in mGy) and DLP (in mGy.cm) values in group B were markedly lower compared with group A. The CTDI_vol_ value in group C was 1.76 ± 0.13, which is significantly lower than 4.22 ± 0.50 of group A (p < 0.001) and 3.45 ± 0.30 of group B (p < 0.001). The DLP value in group C was 235.07 ± 42.41, also significantly lower than those in groups A and B (Table 4).

**Table 4:** Radiation doses among 3 groups.

	Group A	Group B	Group C
CTDI_vol_ (mGy)	4.22 ± 0.50	3.45 ± 0.30*	1.76 ± 0.13^#^
DLP (mGy.cm)	499.15 ± 79.25	400.22 ± 62.24*	235.07 ± 42.41^#^

CTDI_vol_: volume CT dose index; DLP: dose length product. *P < 0.05 compared to group A; and ^#^P < 0.001 compared to both groups A and B. All data were expressed as mean ± standard deviation, and analyzed by one-way ANOVA with Fisher’s least significant difference post hoc test.

### Arm 2: Ultrasound and CT venography results comparison

There were no differences in age, gender ratio, weight, height or BMI among the 3 groups in this arm of study (data not shown). The results from the 3 CT protocols are: 100-kV with 30% weights, 100-kV with 50% weight and 70-kV with 50% weight discovered 12, 12 and 14 patients with DVTs, respectively. Ultrasound identified 13 patients with DVTs in each of the 100-kV groups but 1 in each group was ruled out by CT scanning. In the 70-kV group 14 patients were diagnosed as having DVTs by ultrasound with 1 ruled out and 1 ruled in by CT scanning.

## Discussion

In the present study, using ASIR-V technique, we compared the effects of a 70 kV protocol and the 100 kV protocol in CT venography of lower limb DVT, and observed that: (1) lower tube voltage significantly increased the attenuation value of the vein but not the DVT’s, facilitating DVT diagnosis; (2) although lower tube voltage increased the image noise, the CNR was actually augmented due to the elevated venous attenuation value; (3) the subjective rating of image quality was not significantly different among all groups; and (4) use of lower tube voltage significantly reduced radiation dose.

Radiation exposure from medical imaging has increased significantly in the past several decades, and remains the largest source of average annual radiation exposure that is under our direct control^23^. It has been shown that medical radiation exposure is associated with the subsequent risk of cancer for both pediatric and adult patients^13,24^, necessitating better managing for dose reduction in medical imaging^25^. A number of strategies including modulation of tube voltage have been explored in CT scanning^25^. Lower extremity DVT is a serious medical condition that can result in death or major disability due to pulmonary embolism or post-thrombotic syndrome, requiring early and accurate diagnosis for prompt and effective intervention. CT venography is the key diagnostic modality for DVT^9^. Using the ASIR-V technique combined with low-tube-voltage, we showed that low voltage dramatically reduced the radiation dose (by 40–50%, Table 4). Thus, transformation of the low-voltage protocol into clinical application would be of great benefit for patients.

Tube voltage has a direct influence on radiation dose. McNitt-Gray estimates that if all other technical parameters are held constant, an increase of voltage from 120 kV to 140 kV on a CT/scanner will result in an increase of radiation dose by 37.5% for the head phantom and 39% for the body phantom^26^. Shen *et al*. summarized studies published from January 2011 to November 2015 that aimed to reduce radiation dose using low-tube-voltage, and discovered that low-tube-voltage was a powerful tool to reduce radiation dose, although most of the reviewed studies were conducted with CT angiography^27^. For example, Wang *et al*. showed a marked reduction in radiation dose with an 80 kV protocol compared with 100 kV and 120 kV settings in coronary CT angiography^28^, and similar results were reported in pulmonary CT angiography^29^. However, few studies have investigated the effects of low-tube-voltage in lower extremity CT venography. Cho *et al*. revealed that 100 kV reduced contrast medium, but not irradiation dose compared with a 120-kV setting^30^. In contrast, other studies have shown that low-tube-voltage significantly reduces radiation dose in lower extremity CT venography^17,31^, which align with our findings.

In addition to reducing radiation dose, another advantage for low-tube-voltage is that it improves vascular enhancement. In this study, the 70 kV protocol dramatically increased venous attenuation values while the attenuation values for DVT among different protocols were similar, making it easier for radiologists to recognize DVT, which facilitates the diagnosis. We compared the diagnostic results from ultrasound scanning with those of venography in another arm of study. While each CT protocol ruled out a positive case from ultrasound examinations, the 70 kV protocol additionally identified an ultrasound-determined negative case to be positive. These results are in agreement with reports in the literature, i.e., CT venography is superior to ultrasound in the detection of lower extremity DVTs^9^. The 70 kV protocol corrected a false negative case in ultrasound scanning, likely a result of low voltage leading to increased difference between venous and DVT attenuation values which facilitates DVT diagnosis. Taken together, the low-voltage protocol improves DVT diagnosis, which however needs to be further validated in a large series. Previous studies of CT venography for lower limb DVT also show that low-tube-voltage increases venous enhancement^17,30,31^. Reduction of tube voltage leads to an increase in tube current. Hence, in this study, all protocols were set with an automated tube current, which has been shown to reduce image noise^32^. It has been shown that voltage reduction increases image noise^17,30,31^ and indeed we found group C had significantly higher noise than groups A and B at all measured segmental veins. Nevertheless, the contrast-to-noise ratio in group C was markedly elevated due to enhanced venous attenuation. Furthermore, subjective rating of images from all groups by 2 experienced radiologists did not differ significantly, indicating that 70 kV combined with the ASIR-V technique does not compromise image quality. In view of the results in radiation reduction, image quality and diagnostic accuracy, we concluded that the 70 kV protocol is superior to 100 kV protocols in the detection of lower extremity DVTs.

MBIR is superior to ASIR with regard to reduction of radiation dose and image noise. However, image reconstruction by MBIR takes about 1 h/case, substantially limiting its clinical application^33^. As a new generation of ASIR, ASIR-V, according to the developer, reduces radiation dose up to 82%, improves low-contrast detectability up to 135% and reduces image noise up to 84% relative to FBP (http://www3.gehealthcare.co.uk/~/media/downloads/uk/product/computed-tomography/general/ct%20-%20revolution%20evo%20asir-v%20white%20paper.pdf). A distinctive feature of ASIR-V is that it significantly reduces radiation dose, therefore a higher blending percentage of ASIR-V will result in lower radiation dose, which explains the differences between radiation doses seen between groups A and B. To our knowledge, our study is the first to investigate the effects of the ASIR-V technique in CT venography of lower extremity DVT. Our data show that ASIR-V in combination with 70 kV reduces radiation dose while maintaining image quality and enhancing the diagnostic value, suggesting this technique might be useful for the evaluation of lower extremity DVT in clinical practice.
